# Exploring pathways to recovery and psychological well-being: examining the role of empathic and social self-efficacy, social support and social isolation

**DOI:** 10.3389/fpsyg.2025.1552827

**Published:** 2025-03-17

**Authors:** Maribel G. Dominguez, Louis D. Brown

**Affiliations:** School of Public Health, The University of Texas at Health Sciences Center at Houston, Houston, TX, United States

**Keywords:** peer workers, social support, social isolation, empathic social self-efficacy, substance use, recovery, psychological well-being

## Abstract

**Background:**

This study examines pathways that promote psychological well-being (PWB) and recovery among mental health peer workers. Social support and social isolation are well-established predictors of PWB and recovery. One promising pathway extending from this foundation is that by building empathic and social self-efficacy, individuals can build stronger relationships, which improves social support and reduces social isolation, thereby contributing to recovery and PWB.

**Methods:**

To test this hypothesis, we collected survey data from 268 peer workers on these constructs. We performed a continuous variable mediation analysis to predict recovery and PWB. We examined the direct and indirect effects of empathic and social self-efficacy (ESSE), with social support and social isolation as mediators in pathways toward recovery and PWB.

**Results:**

The direct effect of the ESSE on recovery (B = 0.30 [0.19, 0.42], *p* < 0.001) and PWB (B = 0.26 [0.15, 0.37], *p* < 0.001) was larger than the mediation effects that existed for social support when predicting PWB (B = 0.12 [0.06, 0.20], *p* < 0.001) and recovery (B = 0.11 [0.05, 0.19], *p* < 0.001). Similarly, the direct effect of social support when predicting ESSE on recovery (B = 0.36 [0.25, 0.48], *p* < 0.001) and PWB (B = 0.32 [0.20, 0.43], *p* < 0.001) was larger than its indirect effect through social isolation for both recovery (B = 0.17 [0.11, 0.24], *p* < 0.001) and PWB (B = 0.17 [0.12, 0.24], *p* < 0.001).

**Conclusion:**

Our findings highlight the importance of ESSE in predicting recovery and PWB beyond what can be accounted for by social support and social isolation.

## Introduction

Recovery and psychological well-being (PWB) are established outcomes in mental health in the context of peer support (White et al., 2020). Peer support workers often emphasize these outcomes over clinical outcomes such as mental health symptoms (Kim and Kweon, 2024). Yet the process by which people can make progress toward recovery and psychological well-being lacks clarity. Understanding this process is particularly important for peer support workers, who are a growing part of the mental health system (White, 2019).

Peer support workers are a unique workforce that emphasizes mutual support to promote recovery (Byrne et al., 2022). Peer support workers use their experiential knowledge to help peers with similar lived experiences while promoting the principles of hope, shared power, and mutuality (Austin et al., 2014; Byrne et al., 2022). Evidence suggests that peer workers positively affect individual mental health and well-being, yet the peer worker population is understudied (Austin et al., 2014; Byrne et al., 2022). This study aims to identify pathways to recovery and psychological well-being within the peer support workforce. Understanding these pathways can inform intervention development efforts, enabling a focus on malleable outcomes that are early in the pathway to recovery.

### Psychological well-being and recovery

PWB and recovery are important goals for peer workers and individuals facing mental health and addiction issues. PWB is a multidimensional construct that includes autonomy, environmental mastery, personal growth, positive relations with others, purpose in life, and self-acceptance (Ryff, 1989; Ryff et al., 2003; Ryff and Keyes, 1995). PWB concepts are associated with recovery, and are aligned with peer workers emphasis on hope, shared power, and mutuality (Anthony, 1993; Austin et al., 2014; Byrne et al., 2022).

Recovery is a multidimensional construct that emphasizes connection, hope, autonomy, and informed choice (Anthony, 1993). The recovery model shifts from an emphasis on psychiatric symptoms to a focus on hope, self-management, and social inclusion in developing a meaningful and satisfying life (Sørly et al., 2022). Recovery is an important mental health outcome as it represents striving to reach full potential rather than simply minimizing symptoms (SAMSHA, 2021). Furthermore, recovery as an outcome may help reduce the stigma associated with individuals with a history of substance abuse and mental health issues (SAMSHA, 2021).

### Social isolation

Social isolation has been described as a phenomenon in which an individual lacks a sense of belonging and is thus deficient in fulfilling quality relationships (Linz and Sturm, 2013). Social isolation has been associated with lacking the sense of agency necessary to develop high-quality, satisfying relationships (Linz and Sturm, 2013). Social isolation acts as a stressor that activates various neurotransmitters in the brain that may lead to anxiety and depression (Mumtaz et al., 2018). Individuals with mental health problems report higher rates of loneliness and isolation, which increases the risk for all-cause mortality and drug relapse (Holt-Lunstad et al., 2017; Johnson et al., 2018). Peer support workers address social isolation through outreach efforts aimed at connecting peers to self-help support groups and other peer support mechanisms (Brown et al., 2024). Given the importance of social isolation for health and as a target for peer support worker interventions, we examine it here as a predictor of PWB and recovery.

### Social support

Social support has been associated with improved quality of life and increased mental health recovery (Christine Kerres and Elliott, 1999). Like social isolation, social support plays an important role in recovery and psychosocial well-being (AAP, 2015). Social support has been described as a buffer against the development of psychological issues in individuals coping with stress or stressful situations (Zimet et al., 1988; Zimet et al., 1990). Peer support interventions such as 12-step groups operate as important promoters of social support (Lookatch et al., 2019). Given the centrality of social support for health, and especially how peer support specialists promote health, this study examines it as a predictor of PWB and recovery (Rivas-Rivero et al., 2020).

### Empathic and social self-efficacy

Empathic self-efficacy and social self-efficacy are newer constructs that may be helpful in understanding how peer workers can promote recovery and psychological well-being. Self-efficacy is the confidence to conduct a specific behavior (Bandura, 1977, 1978, 1997, 2002, 2004; Burke et al., 2009). It is important because a high level of self-efficacy can strengthen agency-originated action (Bandura, 2002). Empathic self-efficacy focuses on interpersonal relationships and the level of confidence to respond to others’ needs or feelings empathically (Di Giunta et al., 2010). Thus, perceived empathic ability may promote persistence 91in developing supportive relationships (Mannarini et al., 2017). Empathic self-efficacy also improves individual attitudes toward mental health problems by reducing prejudice and discrimination (Mannarini et al., 2017).

Social self-efficacy is an individual’s belief in their social capabilities and interactions (Kristensen et al., 2022). Previous research suggests social self-efficacy is negatively related to psychological distress (Kristensen et al., 2022). Having the confidence to empathize in a social group is positively related to self-esteem and negatively related to behavior disengagement (Di Giunta et al., 2010). Together, empathic and social self-efficacy may contribute to better relationships, which in turn may lead to better mental health. To our knowledge, previous research has not explored the mediating effect of social support or social isolation as part of the pathway from ESSE to PWB and recovery.

### Current study

**Figure 1:**
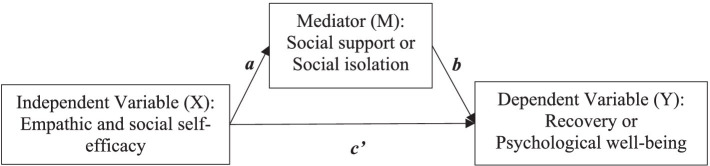
Mediation pathway from ESSE to recovery and psychological well-being.

This study tests hypotheses focused on predicting peer worker recovery and psychological well-being. Specifically, the study examines the direct and indirect effects of empathic and social self-efficacy, with social support and social isolation as mediators in pathways toward recovery and psychological well-being. We have four hypotheses (Figure 1):

1. Social support will mediate the relationship between empathic social self-efficacy and psychological well-being.
2. Social isolation will mediate the relationship between empathic social self-efficacy and psychological well-being.
3. Social support will mediate the relationship between empathic social self-efficacy and recovery.
4. Social isolation will mediate the relationship between empathic social self-efficacy and recovery.

## Method

This cross-sectional study used data from an evaluation of the SHARE! peer toolkit, a training for peer support workers developed by the Self-Help and Recovery Exchange (SHARE!). The purpose of the SHARE! Peer toolkit is to provide training that improves the performance of peer workers in their provision of peer support. The Peer Toolkit covers 12 tools through presentations followed by group discussion and interactive exercises. The Institutional Review Board of the University of Texas Health Science Center at Houston approved the study procedures.

### Data collection

Baseline data was collected from May 2022 to August 2022 via a web-based survey administered to peer workers in the mental health system who agreed to participate. The inclusion criteria for participants (i.e., peer workers) were to be peer workers (i.e., individuals with mental health or substance use lived experience under the mental health system) employed for at least 20 h a week and to have a supervisor at the eligible worksite. The web survey was 15 min long. An initial letter of information was sent to detail the study purpose and any potential risks. Reminder emails, phone calls, and text messages were sent 1-week later and repeated at different times for 2-weeks to encourage completion. The response rate included 694 individuals assessed for eligibility; 306 did not meet inclusion criteria, and 388 met inclusion criteria. However, 268 responded to the baseline data collection.

The data was collected in collaboration with SHARE! The Self-Help and Recovery Exchange. Data cleaning and scoring focused on the primary analytic variables, including social support, empathic and social self-efficacy, social isolation, recovery, and PWB.

### Measures

#### Perceived empathic and social self-efficacy

The empathic social self-efficacy scale is an 11-item 5-point response scale measuring “How well can you…” ranging from 1 = not well at all to 5 = very well. Items 1 to 6 measured empathic self-efficacy, and items 7 to 11 measured social self-efficacy (Di Giunta et al., 2010). The empathic self-efficacy items included questions such as “How well can you read your friends’ needs?” and the social self-efficacy items included “How well can you work or study well with others?.” Due to their high correlation (*r* = 0.91), we treated ESSE as a single scale in this study. The alpha for empathic self-efficacy was α = 0.87, and for social self-efficacy, α = 0.87, the ESSE scale had an α = 0.0.91, indicating good internal consistency. Further, we conducted an exploratory factor analysis (EFA) using the principal factors method with oblique rotation. The analysis indicated that a one-factor solution was appropriate, as the first factor had an eigenvalue of 5.57, while subsequent factors had eigenvalues below 1.

#### Recovery assessment scale-short form

The recovery assessment scale measured was a 24-item scale measuring personal recovery across five domains, including hope, willingness to ask for help, success orientation, reliance on others, and having symptoms under control (Biringer and Tjoflåt, 2018). The measure used a 5-point Likert scale ranging from 1 = strongly disagree to 5 = strongly agree, beginning with the statement, “Following is a list of statements that describe how people sometimes feel about themselves and their lives. Please indicate the response that best describes the extent to which you agree or disagree with the statement.” Questions for the recovery form included “I’m hopeful about my future” and “Even when I do not believe in myself, other people do.” The internal consistency of the overall scale was good at α = 0.82.

#### Psychological wellbeing scale

PWB was 18-items on a 7-point Likert scale ranging from 1 = strongly agree to 7 = strongly disagree (Crouch et al., 2017; Ryff, 1989). The scale asks how the person feels about each statement and it has five subscales, measuring autonomy, environmental mastery, personal growth, positive relations, purpose in life, and self-acceptance. Example items include “I like most parts of my personality” and “Some people wander aimlessly through life, but I am not one of them.” The internal consistency of the overall scale was excellent at α = 0.92.

#### Multidimensional scale of perceived social support

The 12-item scale used 5-point response options ranging from 0 = strongly disagree to 5 = strongly agree with a reliability score of α = 0.88 (Zimet et al., 1988; Zimet et al., 1990). The scale begins with the statement “Indicate how you feel about each statement,” followed by items such as, “I have a special person who is a real source of comfort to me” and “My family really tries to help me.”

#### Social isolation

The Social Isolation—Short Form 6a PROMIS Bank v1.0 is a 6-item scale with five response options, 1 = Never 2 = Rarely, 3 = Sometimes, 4 = Usually, 5 = Always (Ahmad et al., 2019; Measures.Net, 2019). The main scale includes questions such as “I feel isolated from others,” “I feel left out,” and “I feel that people barely know me.” The internal consistency of the overall scale was excellent at α = 0.92.

**Table:** 

Construct	Measurement tool name	Items	Mean	SD
Empathic and social self-efficacy	Perceived Empathic Self-Efficacy (PESE) and Perceived Social Self-Efficacy (PSSE)	11 (1–6, 7–11)	4.29	0.54
Social Support	Multidimensional Scale of Perceived Social Support	12	5.75	1.04
Social Isolation	Social Isolation	6	1.99	0.75
Recovery (hope, willingness to ask for help, personal confidence, goals, and reliance on others)	Recovery Assessment Scale-Short Form	24	4.43	0.48
PWB (autonomy, environmental mastery, personal growth, positive relations, purpose in life, and self-acceptance and environmental mastery)	Psychological Well-being	18	5.78	0.78

### Analysis plan

The Statistical Analysis System (SAS) analytical package was used for all analyses. Before testing hypotheses, we examined the distributions of dependent variables and found that recovery exhibited a skewness of −1.57 and a kurtosis of 7.47. We used a square root transformation to reduce skewness and kurtosis. Post-transformation, recovery had an acceptable skewness of −0.79 and a kurtosis of 1.97. Each hypothesis was tested using the PROCESS macro mediation program (Hayes, 2018). We performed a continuous variable mediation analysis utilizing the PROCESS macro to examine if the mediation variables had an effect on the psychosocial variables. We used bootstrap standard errors and included the covariates of gender, educational attainment, and marital status in all models. Within the analytic sample, missing data was minimal, ranging from 1 to 2% for the variables included. Thus, we used listwise deletion for cases with missing data.

## Results

Among the 268 participants who were eligible to participate, most were female (188, 71.2%), and with some college, had no degree (91, 34.6%), and never married (116, 44.62%; see Table 1). Participant race categories included Black or African American (68, 26.7%), Native Hawaiian or Pacific Islander (1, 0.39%), Asian (14, 5.49%), American Indian or Alaska Native (6, 2.35%), Other (37, 14.51%), Mixed (25, 9.8%) and White (104, 40.8%). The mediation analysis results by path and hypothesis are found in Table 2.

**Table 1:** Demographics.

		*N*	%
Sample Size	Characteristics	268	
Gender	Male	68	25.8
	Female	188	71.2
	Other	8	3.03
Race	White	104	40.8
	Black, African American	68	26.7
	Native Hawaiian or Pacific Islander	1	0.39
	Asian	14	5.49
	American Indian or Alaska Native	6	2.35
	Other	37	14.51
	Mixed	25	9.80
Degree	High School or less, no diploma	12	4.56
	High school graduate; GED; or Equivalent	31	11.79
	Some college, no degree	91	34.60
	Associate degree or technical degree	45	17.11
	Bachelor’s degree (BA, AB, BS, BBA)	66	25.10
	Masters, Doctoral; or Professional degree	18	6.84
Marital status	Never married	116	44.62
	Married	48	18.46
	Divorced	67	25.77
	Widowed	9	3.46
	Separated	7	2.69
	Civil union	13	5.00

**Table 2:** Mediation analysis including, direct, indirect, and total effects of empathic and social self-efficacy (ESSE) on recovery and psychological well-being (PWB).

Model	a path (95% CI)	b path (95% CI)	Indirect effect ab path (95% CI)	Direct effect c’ path (95% CI)	Total effect c path (95% CI)
M = Social support, Y = PWB	0.38 [0.26, 0.49]*	0.31 [0.20, 0.43]*	0.12 [0.06, 0.20]*	0.32 [0.20, 0.43]*	0.43 [0.32, 0.54]*
M = Social isolation, Y = PWB	−0.42 [−0.54, −0.31]*	-0.41 [−0.52, −0.30]*	0.17 [0.12, 0.24]*	0.26 [0.15, 0.37]*	0.43 [0.32, 0.54]*
M = Social support, Y = Recovery	0.37 [0.26, 0.49]*	0.29 [0.18, 0.41]*	0.11 [0.05, 0.19]*	0.36 [0.25, 0.48]*	0.47 [0.36, 0.58]*
M = Social isolation, Y = Recovery	-0.42 [−0.53, −0.31]*	−0.40 [−0.51, −0.29]*	0.17 [0.11, 0.24]*	0.30 [0.19, 0.42]*	0.47 [0.36, 0.58]*

*p < 0.001. X is the independent variable, Empathic and Social Self-Efficacy (ESSE). Y is the dependent variable. M is the mediating variable.

### Effect (X) on the mediator (ΔM)—a-path

The “a” path represents the direct relation between ESSE and the mediator variable (ΔM), which was either social isolation or social support. There was a positive relation between ESSE and social support in hypothesis one (B = 0.38 [0.26, 0.49], *p* < 0.001) and hypothesis three (B = 0.37 [0.26, 0.49], *p* < 0.001). ESSE had a negative relation with the mediating variable of social isolation in hypothesis two (B = −0.42 [−0.54, −0.31], *p* < 0.001) and hypothesis four (B = -0.42 [−0.53, −0.31], *p* < 0.001).

### Effect of (M) to (Y)—b path

The “b” path represents the relation between the mediator (M) and the dependent variable (Y). Social support had a statistically significant positive relation with both outcomes, PWB (B = 0.31 [0.20, 0.43], *p* < 0.001) and recovery (B = 0.29 [0.18, 0.41], *p* < 0.001). In other words, a one-unit increase in social support predicted a 0.31 increase in PWB and a 0.29 increase in recovery. Social isolation, on the other hand, had a negative relation with the outcome variables, PWB (B = −0.41 [−0.52, −0.30], *p* < 0.001) and recovery (B = -0.40 [−0.51, −0.29], *p* < 0.001). In other words, a one-unit increase in social isolation predicted a − 0.41 decrease in PWB and a − 0.40 decrease in recovery.

### Mediating effect—path ab

The “ab” path represented the indirect effect of ESSE on the dependent variable, as mediated by either social support or social isolation. Significant mediation effects existed for both social support and social isolation when predicting PWB and recovery (see Table 2). The effect of the mediators (ΔM) supported hypothesis one (B = 0.12 [0.06, 0.20], *p* < 0.001), hypothesis two (B = 0.17 [0.12, 0.24], *p* < 0.001), hypothesis three (B = 0.11 [0.05, 0.19], *p* < 0.001) and hypothesis four (B = 0.17 [0.11, 0.24], *p* < 0.001).

### Direct effect of (X) to (Y)—c’ path

The c’ path represents the direct effect of ESSE on the dependent variable after accounting for the mediating variable (i.e., represents the effect of ESSE on PWB and recovery after accounting for social support or social isolation). ESSE had a direct effect on PWB after accounting for social support (i.e., hypothesis one; B = 0.32 [0.20, 0.43], *p* < 0.001) and social isolation (i.e., hypothesis two; B = 0.26 [0.15, 0.37], *p* < 0.001). ESSE also had a direct effect on recovery after accounting for social support (i.e., hypothesis three; B = 0.36 [0.25, 0.48], *p* < 0.001) and social isolation (i.e., hypothesis four; B = 0.30 [0.19, 0.42], *p* < 0.001). The magnitude of the direct effect of ESSE on PWB and recovery was larger than the indirect effect through social isolation or social support, as shown in Table 2.

### Total effect—c path

Path “c” represents the total effect, which combines the direct and indirect effects. The total effect of ESSE on PWB was (B = 0.43 [0.32, 0.54], *p* < 0.001). The total effect of ESSE on recovery was (B = 0.47 [0.36, 0.58], *p* < 0.001).

## Discussion

Findings highlight the robust importance of ESSE in its association with recovery and PWB. Although social support and social isolation play an important role in PWB and recovery, they only partially accounted for the variance between ESSE and the outcomes of recovery and PWB. ESSE had a larger direct effect on recovery and PWB, independent of the mediated effects.

### Mediating impact of social support and social isolation

The study by Litt et al. (2005) mediated the relationship between self-efficacy while coping with daily stress and post-traumatic stress symptoms (PTSD), finding that social support was negatively associated with PTSD symptoms. Social support in the form of family function had a significant direct impact on the quality of life (Kekwaletswe et al., 2017). Increased social support decreased depression symptoms significantly (Kekwaletswe et al., 2017). Concordant with our results, social support has been associated with increased empathetic regard in the individual (Davidson et al., 2012).

Previous research suggests pro-social behaviors (including investing in relationships and connection with others) improves self-worth and social connectedness while reducing depressing and anxiety (Taylor et al., 2017). One key implication of our findings is that the peer workforce may be able to enhance PWB and recovery through training focused on increasing empathic and social skills. Although further research is needed, such skill development should promote empathic and social self-efficacy. Our findings suggest that with improved self-efficacy, participants can improve social support, reduce isolation, and strengthen recovery pathways (Davidson et al., 2012).

Our study found that isolation has a stronger relation to recovery and PWB than social support. This may be due to isolation increasing the feeling of loneliness or hopelessness that can hinder the recovery process. Thus, addressing a sense of isolation may be a more effective means of promoting mental health recovery and psychological well-being.

### Theoretical and practical implications

This mediation analysis sheds light on the theoretical implications of the peer worker model, where the sharing of experiential knowledge, along with the fostering of mutual respect and empowerment, can effectively support others facing similar challenges (Kim and Kweon, 2024; SAMHSA, 2024). The findings of this mediation analysis have practical implications in the methods of the peer workforce. Specifically, peer workers may benefit from focusing on building empathic and social skills to strengthen the recovery of individuals facing addiction and mental health problems. Empathy helps an individual understand another individual’s world and experiences, creating a reciprocal understanding and building the foundation for support, decision, and recovery (Mead et al., 2001; Shalaby and Agyapong, 2020).

Our findings suggest that the belief in our ability to effectively understand and navigate social interactions is important for mental health. Perceived empathic and social self-efficacy helps increase the likelihood of recovery through mutual support (Litt and Kadden, 2015; Litt et al., 2005; Park and Sprung, 2015; Zvolensky et al., 2018). Further, empathy is a possible foundation for recovery-oriented practices at the interpersonal level (John, 2023). Developing empathic skills may be foundational in helping people build strong relationships that promote PWB. Peer support and the sharing of experiential knowledge in self-help support groups and other mediums may be an effective strategy for developing empathic skills.

### Strengths, limitations, and future directions

This study used established measurement scales, which increased the validity of the analysis. Additional strengths include a substantial sample size, which helps to provide more precise estimates. Peer workers are an important part of the mental health system; thus, studying pathways to recovery in this sample is useful. However, the limitations of this mediation analysis exist. Generalizability may be limited to peer support workers as it’s not clear if the same pattern of results would be found in other samples. In addition, empathic and social self-efficacy may be more central to the well-being of peer workers relative to the general population, given the importance of ESSE for peer worker positions. Further, causal inference is limited, given that the data is cross-sectional. A future direction following this mediation analysis is the further exploration of the ESSE construct via a latent variable analysis. This will help estimate the possible measurement error of the complex construct, which may help improve practice by providing a better understanding of how empathic self-efficacy and social self-efficacy are related to one another. Given the limited research literature in this area, there is a need for further research to establish the replicability of our findings.

### Conclusion

While extensive research has demonstrated the importance of social support and social isolation, our findings highlight the importance of empathic and social self-efficacy in promoting recovery and PWB. Thus, interventions that enhance ESSE may also impact recovery and psychological well-being. To the best of our knowledge, these pathways between ESSE, social support, and mental health have not been previously explored. ESSE may be an important process by which peer support promotes mental health. Future research can examine whether the sharing of experiential knowledge strengthens ESSE. Our study highlights the value of incorporating ESSE as a construct that adds nuance to understanding peer workers and the road to recovery.

## Data Availability

The original contributions presented in the study are included in the article/supplementary material, further inquiries can be directed to the corresponding author.
